# Navigating Life With Parkinson's Disease: A Focus Group Study on Coping Strategies and Considerations for Self‐Management Support

**DOI:** 10.1111/jan.16414

**Published:** 2024-09-03

**Authors:** Maud M. J. Daemen, Bouke A. A. G. De Bruijn‐Heijligers, Colin Van Der Heijden, Lizzy M. M. Boots, Mayke Oosterloo, Marjolein E. De Vugt, Annelien A. Duits

**Affiliations:** ^1^ Department of Psychiatry and Neuropsychology/Alzheimer Center Limburg, Mental Health and Neuroscience Research Institute Maastricht University Maastricht The Netherlands; ^2^ Department of Medical Psychology Radboud University Medical Center Nijmegen The Netherlands; ^3^ Department of Neurology Maastricht University Medical Center Maastricht The Netherlands; ^4^ Department of Medical Psychology Maastricht University Medical Center Maastricht The Netherlands

**Keywords:** coping, daily life, Parkinson's disease, patient perspectives, phenomenology, quality of life, self‐management, support

## Abstract

**Aim:**

To investigate the experiences of people with Parkinson's disease in coping with and adapting to their disease and to identify considerations for a tailored self‐management support program.

**Design:**

A descriptive phenomenological focus group study.

**Methods:**

Five semi‐structured focus groups were conducted between April 2023 and June 2023 in the Netherlands, with 12 people with Parkinson's disease. Two researchers independently performed an inductive content analysis.

**Results:**

Three principal categories emerged: (1) Rational realisation versus emotional experience: the coping strategy transition. This category includes three main coping strategies: denial or avoidance coping, acknowledging with less active coping and proactive and task‐oriented coping. (2) Factors that influence coping, including mindset and skills, social circles and communication and access to support and care. (3) Considerations for successful self‐management of Parkinson's disease, highlighting key areas such as psycho‐emotional guidance, nutrition and lifestyle, peer support and maintaining autonomy and sense of identity.

**Conclusion:**

Coping and adaptation strategies are individual and dynamic processes, with multiple key or turning points during the transition between strategies. Tailored self‐management support can enhance coping abilities during these transitions, fostering adaptation to a life with Parkinson's disease.

**Implications for the Profession and/or Patient Care:**

A patient‐focused version of an existing blended self‐management support program for family caregivers will be developed, which will be delivered by healthcare professionals.

**Impact:**

This study can help healthcare professionals tailor support for people with Parkinson's disease, emphasising their role in facilitating coping and adaptation. Enhancing self‐management can improve self‐efficacy, quality of life and potentially reduce healthcare utilisation in people with Parkinson's disease.

**Reporting Method:**

Findings are reported according to the COREQ guidelines.

**Patient or Public Contribution:**

Patients and Parkinson's disease experts participated in the preparation and implications of the findings. All participants could contribute to the self‐management support program, either through video interviews or content feedback.

**Summary**

**What does this paper contribute to the wider global clinical community?**

- The study provides insight into dynamic coping strategies, aiding clinicians in understanding and addressing the transition from the rational realisation to emotional experience of having Parkinson's disease.
- Key factors that influence coping strategies are identified, such as mindset, lifestyle and social support, offering a broader clinical perspective for tailored interventions.
- The insights will be used to develop a tailored self‐management program, presenting an approach for clinicians to enhance coping abilities and improve patients' well‐being.

## 1 Introduction

Parkinson's disease (PD) is the most common neurodegenerative movement disorder with both motor and non‐motor symptoms. While PD can be treated, it cannot be cured (Bloem, Okun, and Klein 2021; Cacabelos 2017; Kalia and Lang 2015). Due to its progressive nature, PD has an increasing impact over time on different aspects of life. People with PD often experience loss of work, hobbies, social contacts, role reversal and increased dependency on family‐members, which can have a negative effect on their quality of life (QoL) (Barone, Erro, and Picillo 2017; Cong et al. 2022; Soundy, Stubbs, and Roskell 2014). As such, people with PD must adapt to their limitations in these various domains.

## 2 Background

Early adaptation is paramount in addressing the diverse symptoms and challenges faced by people with PD (Wieringa, Dale, and Eccles 2022). By supporting them in developing coping strategies and adapting to their new situation, they can feel more able to manage their condition and maintain QoL over time (Navarta‐Sánchez et al. 2016). Coping strategies refer to a person's cognitive and behavioural efforts to maintain a state of normalcy when dealing with stressful events (Felton and Revenson 1984) and are related to the psychological status and personality traits (Prins et al. 2023). Active coping is characterised by increased cognitive and behavioural attempts to actively confront the stressful situation and solve the problem, whereas passive coping is associated with inactivity and efforts to avoid or deny the problem.

Furthermore, the progressive course of PD requires a constant process of adjustment, which in turn asks for improving self‐efficacy. Self‐efficacy refers to the belief in one's capabilities to organise and execute the necessary actions to manage prospective situations (Bandura 1977). In the context of living with a chronic disease, it can pertain to one's beliefs about their ability to manage the effects of the disease, handle symptom management, stay engaged in daily routines and adhere to support or treatment options (Chan 2021). Moreover, one's beliefs about their ability to control and influence situations can impact their coping behaviour (Bandura, Freeman, and Lightsey 1999; Bandura 1977). Healthcare professionals can play a role in enhancing self‐efficacy, and helping people with PD cope and adapt to a life with PD. However, professionals with psychosocial expertise are often not considered in the adjustment process. There is a threshold to ask for their support due to the stigma that therapy is for the weak and the uncertainty about the content of the interventions (Duits et al. 2020).

Self‐management support offers a way of helping people with PD play an active role in managing their condition (Milne‐Ives, Carroll, and Meinert 2022). In general, supporting self‐management can improve self‐efficacy and QoL outcomes, and may decrease healthcare utilisation (Tuijt et al. 2020). So far, studies are heterogeneous with promising results for specific self‐management skills and self‐management tailored to specific clinical features and treatment (Pigott et al. 2022), such as the Patient Education Program Parkinson, a self‐management program for people with PD and caregivers in group sessions (A'campo et al. 2010). Interventions traditionally take place in clinical contexts, but there is an increasing body of research supporting the idea that home‐based care can yield comparable clinical outcomes and provide higher levels of service‐user satisfaction (Hall et al. 2023).

An example of a blended self‐management program is the Partner in Balance (PiB) program, which was originally developed for informal carers of people with dementia to help them adapt to their new roles as carers (Boots et al. 2016). The blended aspect of the PiB program allows participants to take part at home, at any time, potentially lowering the threshold for those needing support and providing the desired care. Compared to care as usual and a waiting‐list group, significant improvements in self‐efficacy, QoL and mastery were observed in informal carers of people with dementia who completed the PiB program (Boots et al. 2018). In addition, a recent pilot evaluation of a version of PiB aimed at informal carers of people with PD (Parkinson PiB) demonstrated significant improvements in the self‐efficacy of informal carers (Duits et al. 2021).

The development of a derivative of PiB, specifically tailored to the needs of people with PD themselves, presents a promising opportunity to significantly improve the self‐efficacy of people with PD. To develop such a blended self‐management program for people with PD, it is valuable to first gain deeper insights into the strategies that people with PD use to cope and adapt when facing a chronic and progressive disease. Particularly because this program could ultimately enhance coping abilities and facilitate adaptation to the challenges people with PD encounter.

Hence, in the present qualitative study, we examined the experiences of people with PD in coping and adapting to their disease. Additionally, we evaluated their needs and wishes regarding a support program. These insights will be used to develop a tailored online self‐management program specifically designed for people with PD, effectively creating a patient‐focused version of the Parkinson PiB intervention.

## 3 The Study

### 3.1 Aim

In this study, we aimed to investigate the experiences of people with PD in coping with and adapting to their disease and to identify aspects that need to be considered for the development of a tailored self‐management support program.

## 4 Methods

### 4.1 Design

A descriptive phenomenological approach was used to prioritise the lived experiences of people with PD (Savin‐Baden and Major 2013). Focus groups were chosen as a method because they allow us to gain an in‐depth understanding of the experiences of people with PD regarding how they cope and adapt to their disease, and what their needs and wishes are for a tailored self‐management support program. Furthermore, using focus groups allows participants to respond to each other and discuss topics and related factors (Then, Rankin, and Ali 2014). While focus groups are a valuable method for collecting qualitative data, organising them with people with PD can present certain challenges. The importance of their contribution to research is undeniable, making it crucial to adhere to recommendations to enhance their participation in focus group studies, including: choosing an appropriate location; creating a comfortable environment; limiting group size and allowing time to express and respond to each other (Jones et al. 2021). Our aim was to include 12–15 participants to reach data saturation (Guest, Bunce, and Johnson 2006).

### 4.2 Participants

The study is based on semi‐structured focus groups and the sample comprised of people with PD. Recruitment of participants took place within multiple healthcare organisations involved in PD care in the Netherlands and through the researchers' personal and professional networks in PD care. Convenience sampling was used as an efficient and cost‐effective sampling method. To be included in the study, people with PD were at least 18 years of age, were able to give consent to participate in the study, lived at home and confirmed to have the diagnosis of PD. There were no restrictions on the time since the diagnosis. Prior to participation, people received an information letter, after which they had the opportunity to ask questions to the research team. No participants refrained from participating after receiving the information. The study protocol (non‐waiver) was approved by the Medical Ethics Committee of the Maastricht University Medical Center (#2022–3136), The Netherlands.

### 4.3 Data Collection and Procedure

The focus groups, which included obtaining informed consent, were conducted between April 2023 and June 2023, in the Netherlands. A total of five focus groups were conducted: Two focus groups were held in person and three were held online using Microsoft Teams. In addition, field notes taken during each focus group provided a backup method to record the focus groups' main topics and insights. A semi‐structured topic list was used flexibly to structure the focus groups (Appendix 1). It contained questions related to the impact of PD on daily life, coping strategies, factors that aid in the adaptation process, and considerations for future support that could facilitate coping and adaptation. An iterative process was employed, whereby the content was reviewed and discussed after each focus group. Each focus group was facilitated by an experienced moderator (AAD) who provided guidance throughout the discussions. The moderator was supported by an assistant (MMJD, BAAG or CvdH). All focus groups had an average duration of approximately 90 min.

### 4.4 Data Analysis

Each focus group was audio‐recorded and transcribed verbatim. Independent analysis of the transcripts was carried out by MMJD and BAAG, using the software Atlas.ti. An inductive content analysis was performed to gain a deep insight into the data. This method allows themes and categories to emerge from the data rather than relying on pre‐existing categories (Kondracki, Wellman, and Amundson 2002). To conduct the analysis, MMJD and BAAG first repeatedly read the transcripts and familiarised themselves with the data. Next, open coding was conducted to encompass all aspects of its content. The process of analysis was iterative and comprised multiple analytical sessions in which both authors compared and discussed code structures. After the last session, codes were organised into broader and higher‐order themes, and the transcripts were checked to ensure that all codes were covered within these themes. No codes were established without meaningful data. A mind‐map of these broader themes (Appendix 2) was presented to other researchers within the team. While five focus groups were conducted, the audio recording of one focus group was lost due to technical reasons. Its field notes were used to determine if there were any additions to the results of the analyses from the other focus groups. During a team meeting, the final themes were confirmed and validated, and the authors delved into the underlying patterns and relationships. Consensus was reached among all authors.

### 4.5 Rigour and Reflexivity

All focus groups were facilitated by two people: a moderator and one of the three assistants, to ensure investigator triangulation. They were trained in setting up and conducting qualitative research and possess extensive experience in carrying out focus groups. Their background includes years of clinical experience in PD and research on PD. As the moderator had existing professional relationships with many of the participants, the participants and moderator often already knew each other. The expectation was that this would not impact the focus groups, except that participants might feel more at ease due to their familiarity with the moderator, which could make them more likely to share personal experiences. An iterative process was employed during both data collection and data analysis, involving the review and discussion of content after each focus group, and the data were continually reexamined using insights emergent during analysis. Hence, the iterative process was characterised by a flexible and open approach towards both the process and the topic. Additionally, the data was analysed independently by two authors to ensure analyst triangulation. Checking and validation occurred with additional authors and findings are reported according to the Consolidated Criteria for Reporting Qualitative Research (COREQ) (Tong, Sainsbury, and Craig 2007).

## 5 Results

A total of five focus groups were organised, involving 12 people with PD. In total, 14 individuals were included, but two (one man and one woman) did not participate in the focus groups due to time constraints or personal circumstances. Respectively, each focus group consisted of two, two, three, three and two participants. The average age of the 12 participants was 54.8 years (SD 5.08) and time since diagnosis was 8.8 years (SD 6.55). Eight of the 12 participants were male.

**FIGURE 1:**
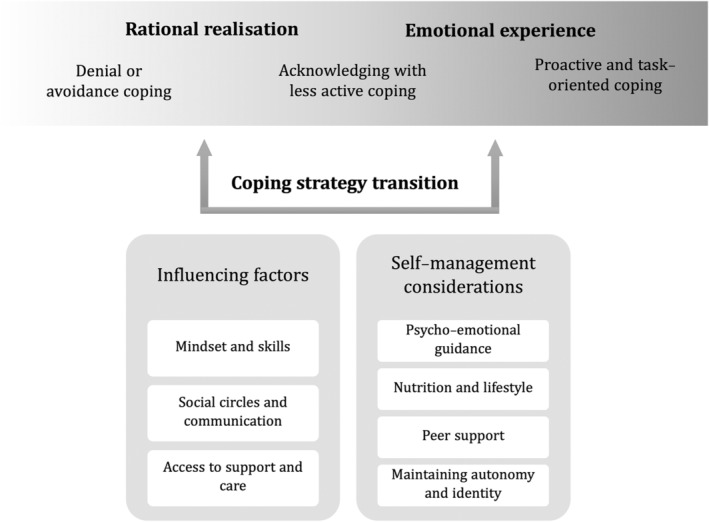
Thematic overview.

The findings portray the challenging and often varying personal experiences of how people with PD cope and adapt to a life with PD. The analysis resulted in three main categories: (1) rational realisation versus emotional experience: the coping strategy transition, (2) coping with PD; influence of mindset, skills, environment and access to care and (3) considerations for successful self‐management of PD. Figure 1 shows the categories and themes.

### 5.1 Rational Realisation Versus Emotional Experience: The Coping Strategy Transition

Participants employ various coping mechanisms to navigate the complexities of PD. Although some strategies may prove more effective than others, individuals often find themselves going through various strategies as the disease progresses. They say going through these different strategies is necessary, due to the discrepancy between the rational realisation of being diagnosed with PD and the emotional experience of having the disease. They described multiple key points during the transition between coping strategies. The sooner they experience these key points, the better their ability to accept and cope with PD.

#### 5.1.1 Denial or Avoidance Coping

Some participants experienced difficulties in accepting their diagnosis or the progression of their symptoms, leading them to deny or pretend as if nothing had happened. These responses were often experienced shortly after receiving the diagnosis or during the early stage of the disease. Additionally, some participants mentioned they exhibited avoidance behaviours, feeling like victims and resisting advice. They developed a sense of fighting against the disease, which could hinder their willingness to seek support or lead to delays in accepting necessary care. Sometimes they felt the need to explore the possibility of other causes being responsible for their symptoms. As a result, they often postponed the start of taking Parkinson medication:

I was totally surprised by the diagnosis. I skipped the processing part. I immediately went on with my daily activities. I just blocked out the news and moved on. Male, focus group 2

I tried so many things. I tried medications to hold on to some hope that it wasn't Parkinson's. When it turned out to be Parkinson's, I didn't even want to tell anyone at work. Male, focus group 3

#### 5.1.2 Acknowledging With Less Active Coping

Overall, participants did not prefer to say they accepted the diagnosis, but gradually, they stopped fighting and resisting against it and acknowledged the disease. Some of them felt a need to learn how to cope with PD, but they also felt reluctant or unsure where to start and how to seek support. There was some hesitancy among them in reaching out for support. They also mentioned that the amount of information was overwhelming and they struggled to gather relevant information. They prefer to have access to tools or resources that can guide them to find information when the need arises, even if it may not be at the current moment:

You need to figure out ways to cope with it yourself and think about who can help you with that. Some people naturally take initiative in that, other may struggle with it. It's these people who could use a helping hand. Male, focus group 4

You need guidance to know where to go or to find information if you search for it yourself. And finding exactly what you need is difficult, so sometimes I tend to say, never mind. Male, focus group 3

#### 5.1.3 Proactive and Task‐Oriented Coping

Most of the participants who had progressed further in their adaptation process showed a proactive approach to seeking support, staying informed about PD and engaging in sharing experiences with others. They consciously made efforts to think in possibilities. It is important to note that this proactive approach didn't imply they were completely free from struggles or challenging situations; rather, they felt better equipped to cope with these difficulties and were more willing to access support or reach out to others. They focused on engaging in activities that brought them energy, acknowledging the importance of self‐care. This active and positive mindset allowed them to better cope with PD and foster a sense of empowerment and resilience:

I engage in more activities again and I express myself more, which has made it easier to talk to people about PD. That allowed me to redirect my focus towards the things I can still do. That has brought me extra positivity and enthusiasm. Female, focus group 3

A fighting spirit arises. You realize that you control your own actions. You must take charge of some things yourself and indicate where you need help. You start living more consciously. I hope others can also experience a positive life with Parkinson's. Male, focus group 4

### 5.2 Coping With PD; Influence of Mindset, Skills, Environment and Access to Care

Participants showed that everyone goes through their own unique adaptation process, which is shaped by various influencing factors.

#### 5.2.1 Mindset and Skills

Participants emphasised the importance of being able to focus on possibilities and positive aspects, such as meeting new people, living more consciously and highlighting the things they can still do, which helped them gain a better and more positive perspective on things. Positive experiences shared by peers also proved to be inspiring, though the seriousness of the disease and people's fear of progression should not be underestimated. They said that life may not be the same as it used to be and they must accept a change within themselves. Adapting the pace, managing their energy level and setting boundaries and priorities is helpful. Many participants found support in maintaining a healthy lifestyle. Activities such as exercising, healthy eating or meditating provided them with a sense of control over their life and body:

The diagnosis felt like a shock. Initially I thought it was all over. As time passed that feeling diminished, and I realized I had to change my mindset. Positive thinking can be learned! Male, focus group 3

#### 5.2.2 Social Circles and Communication

While receiving understanding from the social environment is highly supportive, many participants don't always experience it due to stigmatising beliefs or a lack of knowledge. They encounter incomprehension both within their close circles and from others further away. Most participants only find real understanding among peers. Some participants mentioned that they refrain from sharing or explaining their experiences to avoid burdening others, while the people around them express appreciation when they do share. Relatives also undergo an adjustment process, and effective interaction is key to building understanding. Participants mention that open communication and involving them in their own care process are essential to creating understanding.

I thought I was relieving my partner and children by not burdening them with my struggles. However, now I realize that sharing my experiences grants them insight into the disease and my life, allowing them to be part of the journey alongside me. Female, focus group 1

#### 5.2.3 Access to Support and Care

According to most of the participants, one significant step is learning to seek and accept help. Their coping abilities are influenced by their understanding of available support and knowing where to find it. They mention that coping is an ongoing process, and the need for support fluctuates over time, just as the impact of PD varies from day to day. Individuals handle days differently, some reaching out for assistance promptly while others seek advice. Many of them feel that they have insufficient contact with healthcare professionals, and although they may use certain apps, they don't find accurate support through them. Moreover, they experience limited collaboration among care providers, which drives them to seek help and advice from fellow people with PD as it is a more accessible and comfortable source of support for them:

The neurologist literally shook my hand and wished me good luck. Why is no information given about, for example, sports and PD? It was later, during a cinema break, I saw an advertisement for a sports organization for people with PD. I could not believe that no one had ever mentioned this before. Male, focus group 2

### 5.3 Considerations for Successful Self‐Management of PD

To provide self‐management support, it shows to be important to prioritise resources that are accessible, tailor‐made and adaptive to individual needs. Participants often felt lost and uncertain after they received the diagnosis and emphasised a need for support during this critical period:

Throughout the diagnostic process, I felt support. However, after that, it seemed like I fell into a void of uncertainty – Like I was left on my own. (…). I wish to have support that extends beyond the initial phase. Male, focus group 1

Furthermore, participants noticed an overall focus on pharmaceutical treatment for PD. They expressed varying degrees of criticism or resistance towards this approach. Instead, they professed a preference for more holistic support that includes scientific insights on topics such as nutrition and lifestyle, as well as psycho‐emotional guidance and opportunities for connecting with peers. Participants want to be empowered to learn and utilise these tools independently, without relying on their partner or other family members. By doing so, they can maintain their autonomy and sense of identity, which is highly important to them. Self‐direction and independence are key values and any support, especially self‐management support, should cater to these principles:

The symptoms I experience now belong to me. There's no need for me to fight against them any longer, that is part of who I am now. Living with PD makes you rethink your identity, ‘Who am I in this new chapter with PD?’ Male, focus group 4

## 6 Discussion

In this study, we explored the experiences of people with PD in coping and adapting to their disease and their needs and wishes regarding a support program that could empower them to effectively cope and adapt to the daily challenges they encounter with a progressive disease.

The results gave insight into coping strategies, such as whether and when passive coping was helpful, as well as and how and why strategies may change during the course of PD. Participants have their own unique, adaptation process, which is shaped by their background, mindset, skills, social relationships and access to care, and changes during the disease. They experience multiple key points during the transition between coping strategies, such as avoidance after the PD diagnosis and the development of more active strategies later. It takes time before one is ready to see possibilities instead of only the threat of the disease. People with PD need to be cognitively convinced before they can emotionally open up to the consequences of PD. Based on their needs with respect to support, they want to be empowered to learn in positive thinking and utilise support tools independently, without relying on their partner or other family members. Maintaining autonomy and sense of identity is highly important to people with PD, and they welcome tools that support in their self‐management including peer support and a healthy lifestyle.

Whereas coping is often seen as a stable concept (Stümpel et al. 2022), a recent review in people with PD shows that coping with PD is a dynamic process (Haahr, Groos, and Sørensen 2021), which is in line with our findings. They identified three strategies by which people with PD want to maintain ‘normality’ as long as possible: (1) focusing on the present; (2) staying independent and (3) avoiding challenging situations. To maintain ‘normality’ as long as possible, avoidance and denial can be helpful as also illustrated by the findings of this study. On the other hand, it has been suggested (Hurt et al. 2012) that people with PD who fail to employ task‐oriented coping may be at greater risk of depression, anxiety and poor health‐related QoL. Mild to moderate cognitive impairment may contribute to reduced use of task‐oriented coping. People with PD often use multiple coping strategies in a certain situation (Liebermann, Witte, and Prell 2020) and choice of coping strategies will differ across situations. Age, gender, educational level, anxiety and depression have been found as so‐called determinants of coping strategies in PD (Prins et al. 2023).

The coping strategies emerging from our data were divided into three categories: denial or avoidance coping, acknowledging with less active coping and proactive and task‐oriented coping. An interesting finding is that the participants reported experiencing a key or turning point, when they felt that their coping strategy was not helpful anymore and they needed to make a switch to another strategy. They slowly incorporated PD as part of their life, facing the changes that come with a progressive disease and related losses, but also opportunities and possibilities. This facilitated a more active coping style. This process is in line with the finding that newly diagnosed individuals typically feel disempowered and express a clear need to regain control (Noordegraaf, van den Berg, and Bloem 2023). Insight into the dynamics of coping strategies provides guidance for healthcare providers on when what kind of support may be needed. Understanding turning points and recognising that employing coping strategies, even if it is denial or avoidance coping, is necessary. As participants in our study mentioned, the sooner they experience these key points, the better their ability to accept and cope with PD. Once one is open to possibilities, a positive attitude may help maintain and even increase the perceived quality of life (Albrecht and Devlieger 1999).

In PD, people may experience an upside to living with the disease, which refers to the ability of perceiving ‘silver linings’, including new activities, a healthier lifestyle and improved relationships with relatives and friends (Alonso‐Canovas et al. 2022). This ability to perceive a silver lining of disease fits in with the concept of positive health and underscores the ability to adapt. However, while this is not feasible for all people with PD, its introduction may facilitate the process from feeling lost to regain control (Alonso‐Canovas et al. 2022). The same is true for ‘*hopamine*’, which is a self‐invented neologism of Noordegraaf, van den Berg, and Bloem (2023) for people with a dopamine deficit. Hopamine might offer a tool empowering people with PD to self‐manage their daily life and it stands for the individual's uniquely personal hopes, preferences and abilities. Like the coping strategies described in our study, personal hope is dynamic, individual and context‐driven (Eaves, Nichter, and Ritenbaugh 2016). Hopamine and silver linings can be stimulated by health professionals and are relevant ingredients to support people with PD, especially in the early phase of the disease. This is also related to the concept of positive health, focusing on managing life despite the disease, emphasising a holistic perspective on health (Stiekema, van Heugten, and de Vugt 2019). The participants in our focus groups expressed a need for something other than only medical treatment, including topics such as nutrition and lifestyle, as well as psycho‐emotional guidance and opportunities for connecting with peers. These needs touch those concepts of silver linings and hopamine and this offers a valuable starting point to develop the intended online self‐management tool. Another need of our participants was the need for information and education and the wish to have a central point where they can find all the information they need at that certain point in their PD process. Meeting this need could stimulate a more active and task‐oriented coping strategy.

Building on previous PiB research (Boots et al. 2018; Duits et al. 2021), the intended support program is an online blended application that lowers the threshold for participation as it can be followed at home, at any time and at a suitable pace. It will also have a personalised, modular design with peer‐modelling videos, psycho‐education (information and tips from peers), reflective assignments and personal goal setting. By integrating insights from coping strategies into the content of the program, the program not only provides information and tips, but it also stimulates participants to reflect on their own ways of coping. This reflective process is crucial in helping participants recognise where they stand, understand their own coping strategies and identify areas where they may need further support. This design allows people with PD to find information from peers and to create their personalised ‘hopamine’ recipe. The availability of a version for both people with PD and their care partners offers the possibility of a dyadic approach (Shaffer et al. 2020), from which they may individually benefit, while also mutually enhancing each other positively. The blended approach with a personal and professional coach is helpful to support those with high burden, but also gives the space to work on this independently, which will be highly appreciated given the reported need of maintaining autonomy and sense of identity.

The emphasis on autonomy and identity underscores the importance of incorporating self‐management tools into the support paradigm for people with PD. These findings hold value for policymakers and healthcare professionals, offering insights into the development and provision of tailored support. The proposed online blended support program not only aligns with the evolving technological landscape, but it also provides a response to the growing challenge of healthcare professional shortages. Its remote accessibility positions it as a sustainable option for supporting people with PD. This program can serve as a valuable resource for nursing staff that addresses the specific needs of people with PD.

### 6.1 Limitations

This study has its limitations. The relatively small sample size of this study may impact the generalisability of findings, though data saturation was reached. Also, participants are younger than the average group of people with PD. Coping of younger participants may not accurately reflect those of older individuals with PD, potentially constraining the findings' relevance to the latter group. This could have been mitigated by applying a different sampling method. Convenience sampling is known as a non‐probability sampling method. Additionally, this study employs a single data method, which does not ensure method triangulation. Expanding with observations or objective measures could validate and deepen these findings. Comparing these measures across multiple age groups and between genders would also be valuable. Also, longitudinal studies could enhance our understanding of the evolution of coping strategies throughout the progression of PD.

## 7 Conclusions

This study shows that coping strategies are an individual and dynamic process, while multiple key points during the transition between different strategies are experienced. These phases of coping strategies are influenced by factors such as mindset, social support and access to care. Experienced control and a positive attitude, whilst maintaining being true to oneself, are very important to leading a valuable life with PD. When people with PD come to the point where they need to make changes in their lives due to the progression of PD, they want to be able to find the necessary information and support to do so, including resources related to psycho‐emotional well‐being, nutrition, lifestyle and peer connections. The results underscore the importance of early intervention and tailored self‐management support to facilitate better coping and adjustment to PD. This will empower people with PD to maintain their autonomy and sense of identity, which they consider crucial in their journey with the disease.

## Conflicts of Interest

University Maastricht and the Academic Hospital Maastricht, with Mayke Oosterloo as inventor, have submitted a patent application EP23197746. The other authors report no conflicts of interest.

### Peer Review

The peer review history for this article is available at https://www.webofscience.com/api/gateway/wos/peer‐review/10.1111/jan.16414.

## Data Availability

The data that support the findings of this study are available on request from the corresponding author. The data are not publicly available due to privacy or ethical restrictions.

## Appendix 1

### Semi‐Structured Topic List

**Table:**

Main topic	Subtopics
Living with PD: experiences and coping	What has changed in your life since you have PD? How did you cope with these changes? What has helped you so far to cope with it? What have you missed to be able to cope with it well?
Support strategies	Which elements do you consider important in support for coping with PD? ○ Why? Which topics do you consider important in support for coping with PD? ○ Why? ○ What would you like to know about these topics? For which aspects would you like to receive support in learning to cope with Parkinson's? Do you have any tips on these topics for other people with Parkinson's to help them cope better?

## Appendix 2

### Mind‐Map of Coding Themes

**Table:**

Main theme	Subthemes
Adjusting expectations	Adapting pace Things change and won't be the same as before
Being meaningful	Combining hobbies and activities they have an affinity for, linking to PD Setting new goals
Communication	With healthcare professionals Open communication Patient‐driven, what is the question of the patient Positively framing information Communication to the environment Taking time—preferably less frequent and longer, rather than high frequency and short
Making conscious choices	Setting priorities Setting boundaries
Coping with PD	Denial and avoidance Solution‐oriented thinking Own process, taking control Changes during the process Stop fighting, accept what is, ‘go with the flow’
Dealing with the changing self	Changes in character, appearance, energy level (different pace than before) Preserving identity: who am I, what is valuable to me, how do I shape my ‘new life’ Asking for help when they're not used to it (learning to ask for help) Accepting help
Finding balance	Role patterns within the family Work Daily structure Finding which balance works for me (e.g., activities and what do I value/derive energy from) Setting boundaries and taking breaks Not constantly focusing on PD even though you're confronted with it daily (medication and meal times)
Information provision	Information on ‘other’ topics: alternative treatments, nutrition Searching for information, guidance on where to find it Continuous process
Lack of understanding and knowledge in society	More than before due to media attention Openness/disclosure creates understanding
Lifestyle	Exercise/movement Nutrition Yoga/meditation/mindfulness
Peers	Contact with peers Examples and experiences from others (especially in the early stages) Understanding! They know how I feel Advice
Positivity	Focusing on strengths Activities that give energy and satisfaction PD provides insights, puts things into perspective Positive thinking can be learned Reframing/positively labeling: not a bad day but a rest day
Processing	Patients Family members Each has their own process based on how someone approaches life, influenced by their character and coping style Pro‐active attitude
Scientific insights/fundamental research	Depends on where someone is in the disease process Medication Pesticide Lifestyle
Understanding and knowledge among family members	Need for understanding and attention to psycho‐emotional well‐being Awareness from the patient that family members also go through a process People with PD don't share things to avoid burdening others, while family members indicate that it's nice when they share You don't always have to talk about PD, even though it's present every day. That doesn't mean someone is avoiding or denying it Specific explanation for children (e.g., games and influencers)
Work	Adjustments at work Administrative matters related to the work process
